# Evaluation of collateral status and outcome in patients with middle cerebral artery stenosis in late time window by CT perfusion imaging

**DOI:** 10.3389/fneur.2022.991023

**Published:** 2022-09-13

**Authors:** Mengke Ban, Xue Han, Wanli Bao, Hongli Zhang, Ping Zhang

**Affiliations:** Department of Neurology, The First Affiliated Hospital of Xinxiang Medical University, Xinxiang, China

**Keywords:** stroke, CT perfusion (CTP), collateral, middle cerebral artery (MCA), cerebral blood volume (CBV), cerebral blood flow (CBF)

## Abstract

**Objectives:**

Collateral status (CS) is a crucial determinant of outcome in patients with ischemic stroke. We aimed to test whether the cerebral blood volume (CBV) and cerebral blood flow (CBF) based on computed tomography perfusion (CTP) measurements can quantitatively evaluate CS and explore the predictive ability of CTP parameters in determining clinical outcomes in patients with MCA severe stenosis or occlusion presenting beyond 24 h.

**Materials and methods:**

In this retrospective study, data obtained from September 2018 to March 2022 in consecutive stroke patients caused by isolated middle cerebral artery severe stenosis or occlusion were reviewed within 24–72 h after onset. Correlation between the collateral score systems assessed with CT angiography (CTA) and CTP parameters was calculated using the Spearman correlation. The optimal threshold of the CBV ratio for predicting a good outcome was determined using receiver operating characteristic curve (ROC) analysis.

**Results:**

A total of 69 patients met inclusion criteria. Both the CBV ratio and the CBF ratio had significant correlation with collateral score systems assessed with CTA [CBV ratio and Tan score: r_s_ = 0.702, *P* < 0.0001; CBV ratio and regional leptomeningeal collateral (rLMC) score: r_s_ = 0.705, *P* < 0.0001; CBV ratio and Miteff score: r_s_ = 0.625, *P* < 0.0001. CBF ratio and Tan score: r_s_= 0.671, *P* < 0.0001; CBF ratio and rLMC score: r_s_ = 0.715, *P* < 0.0001; CBF ratio and Miteff score: r_s_ = 0.535, *P* < 0.0001]. ROC analysis revealed the CBV ratio performed better than the qualitative collateral assessments and the CBF ratio in the prediction of a favorable 90-day modified Rankin scale score. The CBV ratio was a useful parameter that predicted a good functional outcome [area under the curve (AUC), 0.922; 95% CI, 0.862 ± 0.982].

**Conclusions:**

In late time window stroke patients, the CBV and CBF ratio on CTP may be valuable parameters for quantitatively revealing the collateral status after stroke. In addition, the CBV ratio was the predictor of clinical outcomes in patients with MCA severe stenosis or occlusion.

## Introduction

Ischemic cerebrovascular disease, characterized by high disability and mortality rates, has increasingly threatened human health (1). Intracranial large-artery atherosclerotic stenosis is the major contributor to ischemic stroke. Especially middle cerebral artery (MCA) stenosis is most common, which may lead to a decrease in cerebral perfusion and the occurrence of ischemic stroke (2). The establishment of collateral status (CS) is closely related to the prognosis of patients with MCA stenosis. Good collateral status may sustain perfusion in the ischemic area, increasing regional cerebral blood flow, saving more extensive penumbral tissue, resulting in smaller final infarction volume, and improving good functional outcomes (3–5). Several qualitative scales have been developed for assessing collateral status on computed tomography angiography (CTA) or Digital subtraction angiography (DSA) based on the degree of contrast opacification of the MCA branches distal to the occlusion (6, 7). DSA is considered the gold standard for assessing collateral circulation. However, DSA is rarely used in clinical practice because it is an invasive examination with the risk of ionizing radiation exposure. Non-invasive assessment of the collateral can be obtained by CTA. Recently, computed tomography perfusion (CTP) examination has played an increasingly important role in diagnosing and treating of ischemic cerebrovascular disease. CTP examination is valuable to diagnose atypical transient ischemic attack (TIA), to direct the intravenous thrombolysis and endovascular mechanical thrombectomy in acute ischemic stroke (AIS), to assess cerebral flow compensation and collateral circulation distal to chronic cerebral artery stenosis, and to screen the patients with symptomatic cerebral artery stenosis for the therapy of revascularization (8). Therefore, the application of CTP imaging in clinical practice is becoming more widespread. CTP has been used to assess collateral status (9, 10). CT perfusion imaging may contribute to comprehensively evaluating the degree of cerebral ischemia, perfusion, and brain tissue compensation by quantitative analysis of perfusion parameters and qualitative analysis of perfusion pseudo-color map, which provides a shred of reliable evidence for the prediction of clinical prognosis. Leptomeningeal anastomosis (LMA) and neovascularization have been shown to be the main collateral compensation methods when the middle cerebral artery is severely stenosed or occluded (11). Collateral flow plays a vital role in making the ischemic area of brain tissue get different degrees of perfusion compensation, and cerebral circulation dynamics reflect collateral status. Therefore, CTP may quantitatively evaluate the cerebral hemodynamics in the ischemic area to accurately evaluate CS and predict the prognosis of stroke patients.

Among the cerebral perfusion parameters, cerebral blood volume (CBV) is observed to reflect the blood flow reserve capacity of ischemic brain tissue. Cerebral blood flow (CBF) measures blood flow velocity and is considered to be representative of the compensation degree of brain tissue blood flow (12, 13). In this study, the ratios of the CTP-derived CBV and CBF values were calculated by dividing the values of the ischemic lesion by the corresponding values of the contralateral normal region (which we defined as the rCBV and rCBF). Some studies found that the rCBV combined with the hyperfusion intensity ratio may evaluate the collateral status (14–16). The relative CBF volume was considered a precise perfusion-based indicator of collateral status (15, 17). These studies aimed to explore the relationship between collateral and perfusion parameters of AIS patients receiving reperfusion treatment in the early time windows of onset (<6 or 24 h). However, a large gap remains between the status of reperfusion treatment of acute ischemic stroke in China and that in developed countries. In China, the pre-hospital delay (discovery, dispatch, transportation) still accounts for the central part of the time of onset to treatment in patients with stroke, which delays the time for AIS patients to reach the comprehensive stroke center, and misses the time window for perfusion evaluation and revascularization treatment. Therefore, the participants of this study are ischemic stroke patients with MCA lesions who unfortunately missed the time windows of thrombolysis or intravascular treatment. To our knowledge, there are insufficient shreds of evidence to predict the status of collaterals using rCBV or rCBF alone. Especially, this relationship has not been well studied in late time windows (beyond 24 h). Our objective was to explore whether rCBV and rCBF, based on CTP measurements, can quantitatively evaluate CS and their values in predicting the clinical outcome for patients with MCA severe stenosis or occlusion presenting beyond 24 h. We compared the predictive ability of collateral score systems assessed with CTA and quantitative CTP parameters in determining clinical prognosis in patients with MCA stenosis.

## Materials and methods

### Patients

This retrospective single institutional study was performed based on the prospective stroke database. Patients with severe atherosclerosis of unilateral middle cerebral artery admitted to the first affiliated Hospital of Xinxiang Medical College within 24–72 h after onset from September 2018 to March 2022 were included. The inclusion criteria were as follows: (1) Patients met the diagnostic criteria of “Chinese guidelines for diagnosis and treatment of Acute Ischemic Stroke 2018” (18); (2) the onset time was 24–72 h, which did not conform to intravenous thrombolysis or endovascular treatment; (3) severe stenosis or occlusion of unilateral MCA-M1 segment confirmed by CTA, stenosis rate ≥ 70%; (4) one-stop CT angiography and CT perfusion were acquired within 7 days after admission. Patients were excluded if they met the following criteria: (1) poor image quality due to motion artifacts or incomplete acquisition; (2) arterial stenosis or occlusion is caused by other causes (moyamoya disease, arterial dissection, vasculitis, cardiogenic embolism); (3) pre-stroke modified Rankin scale (mRS) score of 2 or greater; (4) contraindications in CTA-CTP examination. This study was approved by the Ethics Committee of the First Affiliated Hospital of Xinxiang Medical University (No. 2020024).

### Data collection

Demographic data, risk factors, blood pressure on admission, laboratory indicators on admission, baseline National Institutes of Health Stroke Scale, stenosis degree of a middle cerebral artery and time from admission to CTA-CTP imaging were recorded.

### mRS scores for stroke

The degree of disability or daily activities dependence of patients before the occurrence of stroke was assessed using mRS within the first 24 h after admission. At 90 days after stroke, mRS scores were evaluated by two trained neurologists blinded to the baseline clinical data. Both face-to-face and telephone were allowed. Patients were dichotomized into groups with good (mRS score ≤ 2) or poor (mRS score > 2) outcomes at 90 days.

### One-stop CTA-CTP inspection

**Figure 1:**
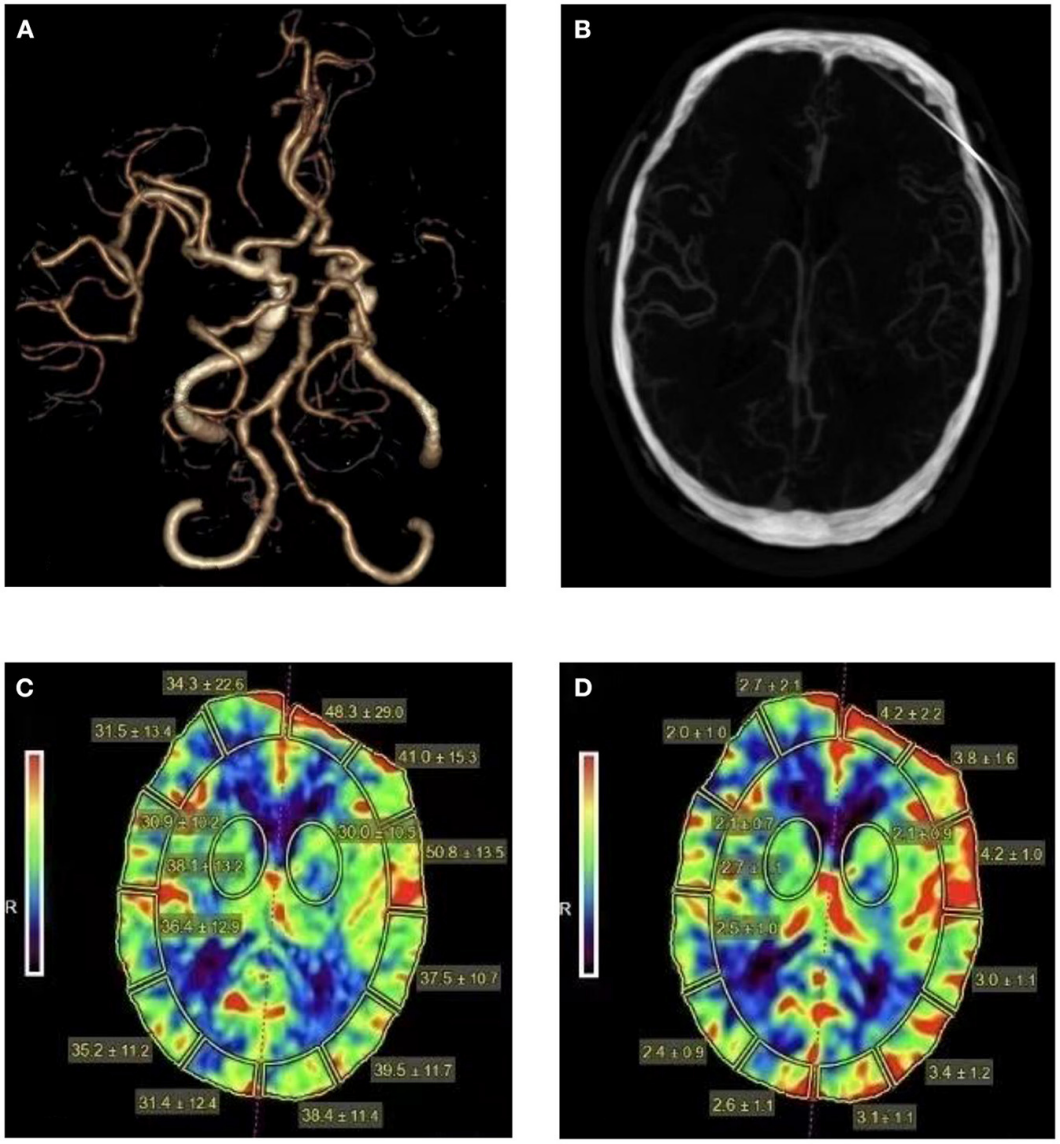
Head CTA, CTA images reconstructed in arterial phase, cerebral blood flow and cerebral blood volume maps for a patient with middle cerebral artery occlusion. A 64-year-old man presented with right limb weakness and was found to have a left MCA stroke with a 90 days mRS of 2 **(A)**. Sufficient collateral vessels can be seen in the occluded MCA territory **(B)**. The CBF **(C)** and CBV **(D)** maps show elevated CBF and CBV of the left hemisphere (CBF ratio = 1.18, CBV ratio = 1.45).

All patients were examined by 320-detector row CT scanner (Aquiliion ONE, Toshiba). Scanning conditions: tube voltage, 80 Kv; tube current, 112 mAs; matrix, 512 × 512; section thickness, 0.5 mm. The coverage was 160 mm. Delayed 5 s after injection of 50 ml contrast agent at a flow rate of 5 ml/s to start dynamic scanning of cerebral perfusion, total scanning time 50 s, interval time 2 s, a total of 25 phases. The CTA images were obtained through motion correction, four-dimensional (4D) noise reduction and automatic bone removal. The middle cerebral artery and the superior sagittal sinus were used as the inflow artery and the outflow vein to generate the time-density curve. After the original parameter data were collected, the data were uploaded to Vitrea Fx software of Toshiba for further analysis, and the software automatically generated pseudo-color maps of CBV, CBF and other parameters. Figure 1 shows the one-stop CTA-CTP images of a patient with middle cerebral artery occlusion.

### Evaluation of cerebrovascular stenosis degree

The rate of cerebral vascular stenosis was assessed according to the standard of the North American Symptomatic carotid endarterectomy trial (NASCET) (19): stenosis rate (%) = (1-Ds/Dn) × 100% (Ds is the diameter of the vessel at the narrowest point of MCA, Dn is the normal diameter of the stenotic vessel). Patients with intracranial vascular stenosis ≥ 70–99% or occlusion were included in the study.

### Collection of cerebral perfusion parameters

Perfusion software automatically delineated the cerebral artery innervation area. Regions of interest (ROI) were defined as bilateral symmetrical brain regions dominated by the cerebral cortex. When the contrast agent passed through the brain tissue, CBF and CBV maps were automatically created according to the time density curve obtained. The mirroring method further measured the corresponding perfusion parameters on the control side. We selected the five layers with the higher time delay on the time to peak (TTP) maps in the middle cerebral artery territory. Then, we calculated the average values of CBF and CBV of the corresponding ROI in the five layers. The rCBF and rCBV were obtained by dividing the values of the affected side by the corresponding values of the unaffected side.

### Qualitative assessment of collateral status

Three qualitative assessment methods were used to evaluate the collateral status by reconstructing CTA images at the artery peak phase. Two neuroimaging physicians with more than 10 years of clinical experience used the blind method to evaluate the collateral circulation, subject to the joint confirmation of the two physicians.

Tan score (20) was used to score according to the filling of the collateral branches of the LMA: 0 point: no collaterals visible; 1 point: the filling of collaterals < 50% of the occluded MCA territory; 2 points: the filling > 50% but < 100%; 3 points: 100% collateral supply. Data were divided into good collateral (score 3), moderate collateral (score 2), and poor collaterals (score 0, 1).

The regional leptomeningeal collateral (rLMC) score (21) was calculated based on the extent of contrast opacification in arteries distal to the occluded MCA. The parasagittal anterior cerebral artery territory, the basal ganglia, the Sylvian sulcus, and the M1-M6 regions of the cortical ASPECTS regions were assessed (22). As follows: non-vascular was seen (0 score), less prominent (1 score), equal or even more prominent compared with the contralateral side (2 scores). Collateral arteries within the Sylvian sulcus scored 0, 2, or 4. The total score was 20, and a higher score indicates a better collateral status.

Miteff score (23) was conducted to score according to the degree of distal vascular reconstruction of the occluded MCA and included three categories: good collateral (3 points): the distal branches of the occluded vessels were reconstructed; moderate collateral (2 points): vessels could be reconstructed within the Sylvian fissure; poor collateral (1 point): the distal superficial branches could be seen.

### Statistical analysis

The categorical variables in this study were presented as the frequency and percentage, and the chi-squared (χ^2^) test was used to compare the differences between the two groups. The continuous variables conforming to normal distribution were reported as the mean and variance, and the data analysis was performed using the *t*-test. While the continuous variables conforming to non-normal distribution were presented as the median and interquartile range using the Mann-Whitney *U* test. The relationship between the rCBV or rCBF and the three qualitative collateral evaluation methods was assessed using Spearman's correlation coefficient and was demonstrated using a scatter plot with a regression line. The receiver operating characteristic (ROC) analyses were performed to assess the predictors of good outcomes for the patients, and the area under the receiver operating characteristic curve (AUC) was calculated. Optimal cutoff values were derived from ROC curves, and the sensitivity and specificity were calculated based on these best cutoff values. In addition, the DeLong test was used to compare the AUC-ROC of four prediction models (CBV ratio vs. the Tan score, CBV ratio vs. the rLMC score, CBV ratio vs. the Miteff score, CBV ratio, and CBF ratio) to determine the best prediction model. Interrater reliability was assessed with the intraclass correlation coefficient (ICC) analysis. All statistical analyses were performed using SPSS software (version 26.0) and MedCalc software (version 19.0). *P* < 0.05 was considered indicative of a significant difference.

## Results

**Figure 2:**
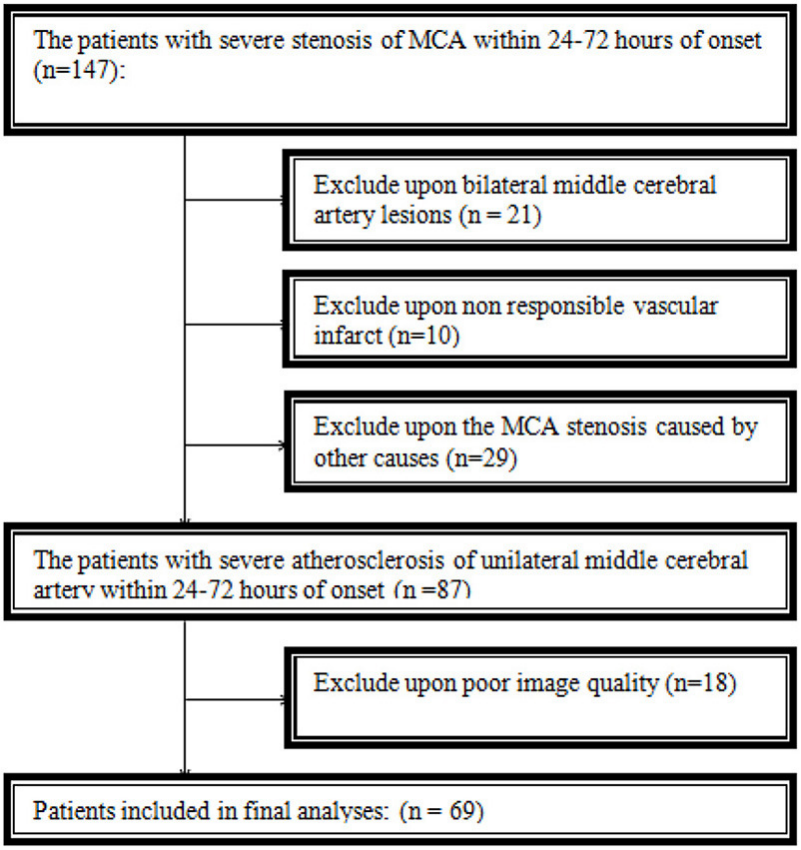
Population flowchart. MCA, middle cerebral artery.

A total of 69 patients were eventually included in this study. Thirty-one patients were excluded because of bilateral middle cerebral artery lesions or non-responsible vascular infarct. Twenty-nine patients were excluded from arterial stenosis or occlusion caused by other causes, and eighteen were excluded because of poor image quality (Figure 2).

### Patient characteristics

The included patients were dichotomized into good (*n* = 38) and poor (*n* = 31) outcomes. Demography, vascular risk factors, blood pressure at admission, laboratory indicators, degree of vascular stenosis and time from admission to CTA-CTP imaging were not significantly different between the good vs. poor outcome groups, except for median baseline NIHSS, which was significantly higher in poor outcome patients (9, IQR = 6–11) compared to good (2, IQR = 1–6) (*P* < 0.01). The baseline data of all patients were summarized in Table 1. The two groups showed significant differences in CBV ratio, CBF ratio, Tan score, rLMC score, and Miteff score. Median or mean baseline CBV ratio, CBF ratio, Tan score, rLMC score, and Miteff score were significantly lower for poor outcome patients than good (Table 2).

**Table 1:** Patient baseline demographic and medical characteristics.

**Variable**	**Good outcome (*n* = 38)**	**Poor outcome (*n* = 31)**	***t*-value, *Z*-value or *χ^2^* value**	***P*-value**
**Demographic data**
Age (years, mean ± SD)	55 ± 8.868	61 ± 14.125	−1.905	0.063
Men (n, %)	29 (76.3%)	20 (64.5%)	1.155	0.283
**Risk factors (** ***n*** **, %)**
Hypertension	24 (63.2%)	20 (64.5%)	0.014	0.907
Hyperlipidemia	2 (5%)	1 (3.2%)	–	1^a^
Diabetes mellitus	7 (18.4%)	6 (19.4%)	0.01	0.921
Coronary heart disease	2 (5.2%)	4 (12.9%)	0.477	0.49
History of previous stroke or TIA	9 (23.7%)	9 (29%)	0.253	0.615
Current smoking	19 (50%)	16 (51.6%)	0.018	0.894
Current drinking	14 (36.8%)	10 (32.3%)	0.158	0.691
**Blood pressure (mmHg, mean** **±SD)**
Systolic blood pressure	140.34 ± 16.78	147.32 ± 25.78	−1.355	0.18
Diastolic blood pressure	85.11 ± 12.52	83.78 ± 10.76	0.468	0.642
**Clinical parameters**
TC (mmol/L, mean ± SD)	3.89 ± 1.04	4.24 ± 1.17	−1.316	0.193
TG (mmol/L, median, IQR)	1.24 (0.91–1.84)	1.19 (0.93–1.79)	−0.229	0.819
LDL-C (mmol/L, mean ± SD)	2.21 ± 0.75	2.35 ± 0.86	−0.69	0.492
HDL-C (mmol/L, median, IQR)	1.02 (0.94–1.46)	1.19 (0.99–1.36)	−1.768	0.077
Glucose (mmol/L, mean ± SD)	5.34 ± 1.29	5.3 ± 1.43	0.11	0.913
Scr (μmol/L, median, IQR)	57.5 (51.73–73.38)	54 (46.2–63)	−1.828	0.068
Hcy (μmol/L, median, IQR)	15.0 (9.31–19.85)	15.54 (10.23–23.72)	−0.718	0.473
Baseline NIHSS score (median, IQR)	2 (1–6)	9 (6–11)	−5.017	<0.01
Stenosis degree of MCA (*n*, %)			0.957	0.328
Severe stenosis	14 (37%)	8 (25.8%)	–	–
Occlusion	24 (63%)	23 (74.2%)	–	–
Time from admission to CTA-CTP imaging (hours, median, IQR)	49.0 (42.75–70.75)	51.0 (46.0–68.0)	−0.440	0.660

^a^Continuous correction chi square test; TIA, transient ischemic attack; TC, total cholesterol; TG, triglyceride; LDL-C, low-density lipoprotein cholesterol; HDL-C, high-density lipoprotein cholesterol; SCr, serum creatinine; Hcy, homocysteine; NIHSS, National Institutes of Health Stroke Scale; MCA, middle cerebral artery.

**Table 2:** Comparing CTP parameters and qualitative collateral scores between patients with good and poor outcomes.

**Variable**	**Good outcome (*n* = 38)**	**Poor outcome (*n* = 31)**	***t-*value or *Z*-value**	***P*-value**
CBV ratio (median, IQR)	1.114 (1.08–1.16)	0.93 (0.88–1.06)	−5.981	<0.01
CBF ratio (mean ± SD)	0.947 ± 0.123	0.824 ± 0.108	4.361	<0.01
Tan score (median, IQR)	2 (1.75–3)	1 (1–2 )	−3.217	<0.01
rLMC score (median, IQR)	14 (12–18)	10 (9–12)	−5.031	<0.01
Miteff score (median, IQR)	2 (1–3)	1 (1–2)	−3.021	<0.01

### Relativity analysis

**Figure 3:**
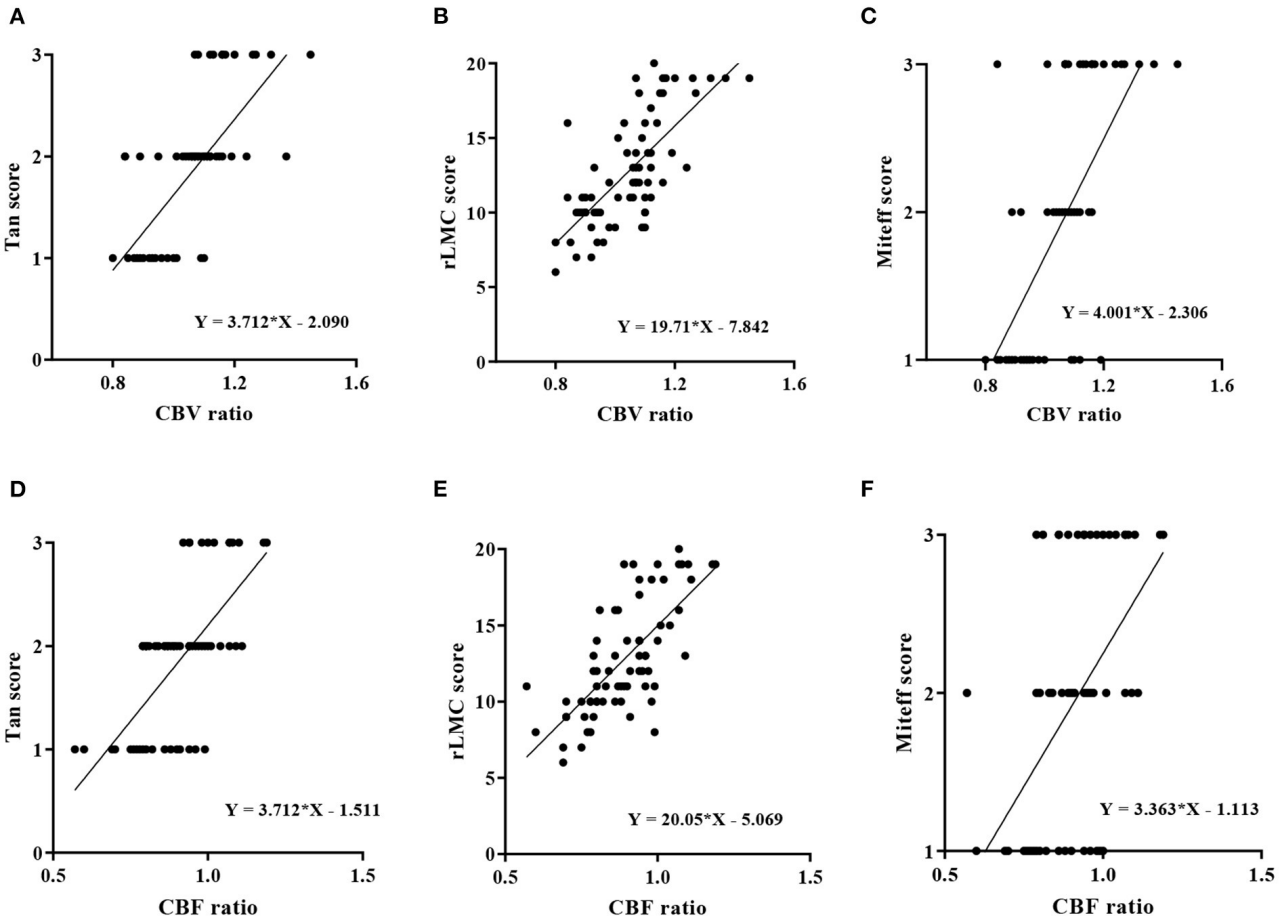
Correlation analysis of perfusion parameters and the three qualitative assessments of CS. **(A)** rCBV was strongly positively correlated with the Tan score (r_s_ = 0.702, *P* < 0.0001). **(B)** There was a strong positive correlation between the rCBV and rLMC score (r_s_ = 0.705, *P* < 0.0001). **(C)** The rCBV and Miteff score were strongly positively correlated (r_s_ = 0.625, *P* < 0.0001). (**D)** The rCBF and Tan score were strongly positively correlated (r_s_ = 0.671, *P* < 0.0001). **(E)** There was a strong positive correlation between the rCBF and rLMC score (r_s_ = 0.715, *P* < 0.0001). **(F)** The rCBF and Miteff score were moderately positively correlated (r_s_ = 0.535, *P* < 0.0001).

The correlations between the CBV ratio, CBF ratio and the three qualitative assessments of CS among the 69 patients were shown using scatter plot with regression line. The CBV ratio showed a strong correlation with Tan score, rLMC score and Miteff score (CBV ratio and Tan score: r_s_ = 0.702, *P* < 0.0001, Figure 3A), (CBV ratio and rLMC score: r_s_ = 0.705, *P* < 0.0001, Figure 3B), (CBV ratio and Miteff score: r_s_ = 0.625, *P* < 0.0001, Figure 3C). The CBF ratio showed a strong correlation with Tan score, rLMC score and Miteff score (CBF ratio and Tan score: r_s_= 0.671, *P* < 0.0001, Figure 3D), (CBF ratio and rLMC score: r_s_= 0.715, *P* < 0.0001, Figure 3E), (CBF ratio and Miteff score: r_s_ = 0.535, *P* < 0.0001, Figure 3F).

### Collateral assessment as a predictor of good outcome

**Figure 4:**
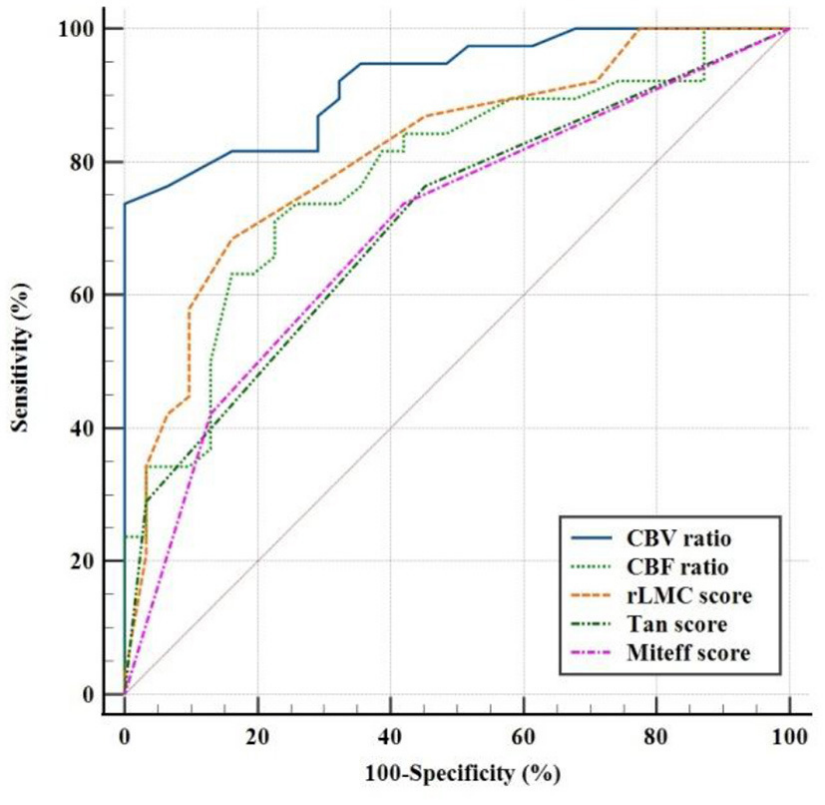
Receiver operating characteristic curves showed sensitivity and specificity in predicting a good clinical outcome with three qualitative assessments of CS and CTP predictors.

ROC analysis was used to evaluate the CBV ratio and other qualitative collateral assessments as predictors of a good outcome. In terms of predicting good functional outcome, the CBV ratio (AUC = 0.922; 95% CI, 0.862 ± 0.982) performed better than rLMC score (AUC=0.815; 95%CI, 0.761 ±0.944), CBF ratio (AUC = 0.779; 95% CI, 0.669 ± 0.889), Tan score (AUC = 0.709; 95% CI, 0.587 ± 0.830), Miteff score (AUC = 0.699; 95% CI, 0.575 ± 0.824). The optimal threshold of the CBV ratio for predicting good functional outcomes was 1.085 (sensitivity = 73.7%, specificity = 100%). Specific AUC related values for each variable were shown in Table 3 and were demonstrated in Figure 4. The pairwise comparison of ROC-AUC by DeLong test of MedCalc software revealed that there were significant differences (CBV ratio vs. rLMC score: *Z* = 2.116, *P* = 0.0343), (CBV ratio vs. CBF ratio: *Z* = 2.304, *P* = 0.0212), (CBV ratio vs. Tan score: *Z* = 4.725, *P* < 0.0001), (CBV ratio vs. Miteff score: *Z* = 4.254, *P* < 0.0001).

**Table 3:** The area under curve, sensitivity, specificity, and optimal threshold of CTP predictors and qualitative assessments of CS.

**Variable**	**AUC (95% CI)**	***p***	**Optimal threshold**	**Sensitivity**	**Specificity**
CBV ratio	0.922 (0.862–0.982)	<0.01	1.085	73.7%	100%
CBF ratio	0.779 (0.669–0.889)	<0.01	0.895	71.1%	77.4%
rLMC score	0.852 (0.761–0.944)	<0.01	12.5	71.1%	83.9%
Tan score	0.709 (0.587–0.830)	<0.01	2	76.3%	54.8%
Miteff score	0.699 (0.575–0.824)	<0.01	2	73.7%	58.1%

### Reproducibility assessment

Since cerebral perfusion parameters were automatically generated by Perfusion software, there was no interobserver error in calculating the CBV and CBF ratios. Excellent interobserver agreement was detected in qualitative assessments of CS (ICC = 0.94, 0.95, and 0.96 for Tan score, Miteff score, and rLMC score, respectively).

## Discussion

This study found that rCBV and rCBF based on CTP were significantly associated with qualitative collateral assessments. Regarding the short-term outcome, compared with the rCBF and other collateral assessment methods, the rCBV was a predictor of good functional outcomes for patients with middle cerebral artery stenosis or occlusion.

CTP is a superior imaging technique that contributes to evaluating the state of cerebral blood perfusion, showing the changes in cerebral perfusion parameters in the ischemic stroke lesions and effectively reflecting cerebral ischemia and collateral circulation in the early stage of the disease (9, 24). CT perfusion examination plays an increasingly important role in diagnosing and treating of ischemic cerebrovascular disease. In order to determine whether CTP parameters can evaluate the collateral status and predict short-term outcomes in patients with MCA stenosis or occlusion presenting beyond 24 h, 69 patients were dichotomized into good outcomes and poor outcomes according to the recovery of neurological function 3 months after onset. There were no significant differences in demographic data, risk factors, blood pressure on admission and biochemical indicators between the two groups. However, the group with a good prognosis had a lower NIHSS score and better collateral circulation. NIHSS score is a vital scale to evaluate the neurological impairment and prognosis of stroke patients, and it has been widely used in the clinic.

It has been reported to use cerebral perfusion parameters to evaluate collateral status. Shi et al. (25) have suggested that measuring the maximum cerebral blood flow of collateral vessels within the Sylvian fissure was a feasible quantitative collateral assessment at perfusion CT. Park et al. (26) have revealed a strong correlation between rCBV and the collateral flow grade on DSA after AIS. Some studies have shown that rCBV combined with a lower hypoperfusion intensity ratio may be a useful diagnostic tool for evaluating the collateral status (14, 16). However, the automatic perfusion evaluation software for calculating mismatch volume is required. Advanced perfusion analyzers are not introduced in most stroke centers in China, so the quantitative evaluation of the collateral circulation method is more suitable for practical clinical work. The ability to automatically derive an objective measure of collateral status using CTP may have significant clinical implications for predicting patient outcomes. Our results demonstrated that rCBV and rCBF were positively associated with the qualitative assessments of CS, which may reflect the perfusion of microcirculation and thus may indirectly estimate collateral status. The rCBV and rCBF are quantitative assessments, requiring no additional training for scoring scales. Compared with these CTA scoring systems, there may be significant advantages in using the rCBV and rCBF for collateral assessment. Our study emphasizes the importance of measuring the CBV ratio and CBF ratio on CTP as they provide an easier means for evaluating the extent of the collateral blood flow or residual blood volume in stroke patients. In the event of middle cerebral artery stenosis or occlusion, cerebral perfusion pressure and regional cerebral blood flow may decrease significantly. For those with good collaterals, the maintenance of local residual blood flow and perfusion pressure mainly depends on compensatory dilatation of collateral vessels and retrograde leptomeningeal collateral flow. Perfusion *via* leptomeningeal collateral vessels may preserve brain tissue at risk of infarction in the ischemic border zone (27, 28). Meanwhile, rCBV is unchanged or elevated, and rCBF is unchanged or slightly decreased. For patients with poor collaterals, the continuous decrease of cerebral perfusion pressure exceeds a certain compensatory limit, significantly decreasing the rCBV and rCBF, eventually reducing collateral flow to the penumbra. Therefore, our study supported using rCBV and rCBF to assess collateral in patients with MCA stenosis whose onset is in the late time window.

Functional outcome in stroke patients depends on restoring blood flow in a timely fashion to salvage brain tissue at risk, thereby reducing the extent of the final infarct (29). The CBV ratio was considered to reflect the development of the collateral circulation and microcirculatory vascular bed. During severe vascular stenosis or occlusion of M1 segment MCA, the compensatory dilatation of collateral and microcirculatory vessels plays a significant role in increasing the blood supply in the MCA territory infarction and enhancing perfusion reserve capacity in the lesion territory, which eventually may improve the function of brain cells in the ischemic penumbra, reduce the final infarction size and prevent brain function damage. The functional prognosis of AIS patients is associated with collateral status. The status of collateral blood flow has been found to influence the severity of neurological damage and the size of ischemic stroke lesions (30). Thamm et al. (31) reported that higher quantitative CBF was a significant predictor of a good neurological outcome at day 90. Compared with previous studies, our study confirms that rCBV has better diagnostic efficacy in evaluating the good outcome of patients with MCA stenosis or occlusion. In addition to previously published results which have shown the utility of CBF and CTA in the assessment of collaterals and outcome, our study adds evidence to support including rCBV in the prediction of outcome. On the practical clinical utility of the CBV parameter in stroke patients, Lou et al. (32) reported that higher CBV scores might be a specific marker of clinical outcome in patients with acute MCA ischemic stroke. d'Esterre et al. (33) investigated the CBV images in 55 patients and found a reduction in CBV was a valuable predictor of infarct core. However, our study identified an rCBV threshold of =1.085 as an optimal predictor of a good outcome for patients with MCA. The diagnostic sensitivity and specificity are excellent. Patients with good outcomes had a significantly higher CBV ratio compared with poor outcomes. This finding is in agreement with previous studies (34–36).

The strength of our study is that we focused on the relationship between CTP parameters and collateral and prognosis in the late time window (beyond 24 h), which adds new evidence for collateral and prognosis evaluation in this part of patients with MCA severe stenosis or occlusion. This also limits the interpretation of our conclusions, which may not be suitable for patients in earlier time window (<6 h). Another limitation is that our conclusions are only applicable to patients with unilateral large MCA stenosis or occlusion, and cannot be generalized to ischemic stroke of other vascular territories or caused by other mechanisms and small vessel occlusions, which may reduce the external validity of our study. Another limitation is that no unified method for acquiring perfusion parameters depends on the software at hand. Different perfusion software provides different possibilities for defining the regions of interest. The different acquisition methods of perfusion parameters may bias the research results. Finally, our study was a retrospective observational design using data from a single-center, and the small sample size was inadequate to make definitive conclusions. Therefore, it is necessary to expand the sample size for prospective analysis further to determine the application value of CTP parameters in patients with MCA stenosis.

## Conclusion

In conclusion, in MCA stenosis patients presenting in late time window, good collaterals were significantly associated with greater CBV and CBF ratios. Compared with the CBF ratio and CTA-based methods for evaluating prognosis, the CBV ratio was the better predictor of a good prognosis.

## Data availability statement

The original contributions presented in the study are included in the article, further inquiries can be directed to the corresponding author.

## Ethics statement

The studies involving human participants were reviewed and approved by the Ethics Committee of the First Affiliated Hospital of Xinxiang Medical University (No. 2020024). The patients/participants provided their written informed consent to participate in this study.

## Author contributions

MB contributed to the conception of the study, methodology, investigation, formal analysis, and writing—original draft. XH performed the data curation and wrote the manuscript. WB and HZ contributed significantly to review and check data. PZ helped perform conceptualization, funding acquisition, resources, and supervision. All authors contributed to the article and approved the submitted version.

## Funding

This study was supported by 2019 Joint Construction Project of Henan Provincial Health Committee and Ministry of Health (SB201901061), Henan Key Laboratory of Neurorestoratology (Grant No. HNSJXF-2021-004), and Postgraduate Education Reform and Quality Improvement Project of Henan Province (YJS2021AL063).

## Conflict of interest

The authors declare that the research was conducted in the absence of any commercial or financial relationships that could be construed as a potential conflict of interest.

## Publisher's note

All claims expressed in this article are solely those of the authors and do not necessarily represent those of their affiliated organizations, or those of the publisher, the editors and the reviewers. Any product that may be evaluated in this article, or claim that may be made by its manufacturer, is not guaranteed or endorsed by the publisher.
